# BMAL1 Promotes Valvular Interstitial Cells’ Osteogenic Differentiation through NF-κ B/AKT/MAPK Pathway

**DOI:** 10.3390/jcdd10030110

**Published:** 2023-03-06

**Authors:** Yefan Jiang, Song Wang, Wenfeng Lin, Jiaxi Gu, Geng Li, Yongfeng Shao

**Affiliations:** 1 Department of Cardiovascular Surgery, The First Affiliated Hospital of Nanjing Medical University, Guangzhou Road, No. 300, Nanjing 210029, China; 2 Department of Cardiovascular Surgery, Union Hospital, Tongji Medical College, Huazhong University of Science and Technology, Jiefang Road, No. 1277, Wuhan 430022, China

**Keywords:** BMAL1, valve interstitial cells, osteogenic differentiation, calcified aortic valve disease

## Abstract

Objectives: Calcific aortic valve disease (CAVD) is most common in the aging population and is without effective medical treatments. Brain and muscle ARNT-like 1 (BMAL1) is related to calcification. It has unique tissue-specific characteristics and plays different roles in different tissues’ calcification processes. The purpose of the present study is to explore the role of BMAL1 in CAVD. Methods: The protein levels of BMAL1 in normal and calcified human aortic valves and valvular interstitial cells (VICs) isolated from normal and calcified human aortic valves were checked. HVICs were cultured in osteogenic medium as an in vitro model, and BMAL1 expression and location were detected. TGF-β and RhoA/ROCK inhibitors and RhoA-siRNA were applied to detect the mechanism underlying the source of BMAL1 during HVICs’ osteogenic differentiation. ChIP was applied to check whether BMAL1 could directly interact with the runx2 primer CPG region, and the expression of key proteins involved in the TNF signaling pathway and NF-κ B pathway was tested after silencing BMAL1. Results: In this study, we found that BMAL1 expression was elevated in calcified human aortic valves and VICs isolated from calcified human aortic valves. Osteogenic medium could promote BMAL1 expression in HVICs and the knockdown of BMAL1 induced the inhibition of HVICs’ osteogenic differentiation. Furthermore, the osteogenic medium promoting BMAL1 expression could be blocked by TGF-β and RhoA/ROCK inhibitors and RhoA-siRNA. Meanwhile, BMAL1 could not bind with the runx2 primer CPG region directly, but knockdown of BMAL1 led to decreased levels of P-AKT, P-IκBα, P-p65 and P-JNK. Conclusions: Osteogenic medium could promote BMAL1 expression in HVICs through the TGF-β/RhoA/ROCK pathway. BMAL1 could not act as a transcription factor, but functioned through the NF-κ B/AKT/MAPK pathway to regulate the osteogenic differentiation of HVICs.

## 1. Introduction

CAVD is the most common valvular disease, with high morbidity and mortality rates [1]. It results in aortic valve thickening, calcification and eventually, hemodynamic abnormality. Thoracotomy and interventional surgeries are the only effective treatments and no medical therapies have yet been developed [2].

VICs are the most abundant cells in aortic valves. Although valvular endothelial cells take part in the development of CAVD, great evidence has proven that VICs’ phenotype change is the main cause [3,4]. During disease initiation and progression, VICs undergo myofibroblastic and osteogenic differentiation, thereby evoking extracellular matrix remodeling, collagen deposition, nucleation loci formation and eventually, osteoblastic VIC-mediated bone formation [5].

The circadian clock genes regulate the circadian rhythm and maintain normal physiological function; their disorder may result in diseases [6,7]. These clock genes exist in both brain and peripheral tissues, known as central and peripheral clock genes [8]. The central clock genes existing in the suprachiasmatic nucleus (SCN) regulate the circadian rhythm and their disorder may lead to the disturbance of the 24 h light/darkness cycle and eventually, illness [9,10]. The peripheral clock genes can be controlled by both central clock genes and peripheral stimuli. They can regulate the local cell physiology without a disturbed circadian rhythm [11,12]. Clock genes can take part in the calcification process. They are not only related to physiological calcification, such as bone formation and dental development, but also pathological calcification, including vessel and tendon calcification [13,14,15,16,17].

BMAL1 acts as a core clock gene and is necessary for the circadian rhythm [18,19]; it is involved in the calcification process. Runt-related transcription factor 2 (runx2), as the key transcription factor associated with osteogenic differentiation, showed 24 h periodicity in the SCN and bone and its periodicity disappeared in BMAL1-KO mice [20]. BMAL1 participates in physiological calcification; it can affect bone formation, dental development and the osteogenesis of bone marrow stromal cells [21,22,23]. Moreover, it can also contribute to pathological calcification, such as atherosclerosis and mandibular dysplasia [24,25]. BMAL1 can serve as a transcription factor alone or combined with CLOCK to form heterodimers, which can activate the transcriptional activity of downstream genes [24,26,27]. Meanwhile, it can affect the calcification process through the BMP/Wnt/p53 signaling pathway [22,28,29]. BMAL1 shows unique tissue-specific characteristics and plays different roles in the calcification processes in different tissues [30,31]. Its role in CAVD still needs further exploration.

We hypothesized that BMAL1 promoted valve calcification. The purpose of this study is to determine (1) whether BMAL1 accumulation is associated with CAVD; (2) the effect of BMAL1 on HVICs’ osteogenic differentiation; and (3) the mechanism by which BMAL1 exerts its effect.

## 2. Results

### 2.1. BMAL1 Expression Is Increased in Calcified Human Aortic Valves

**Figure 1:**
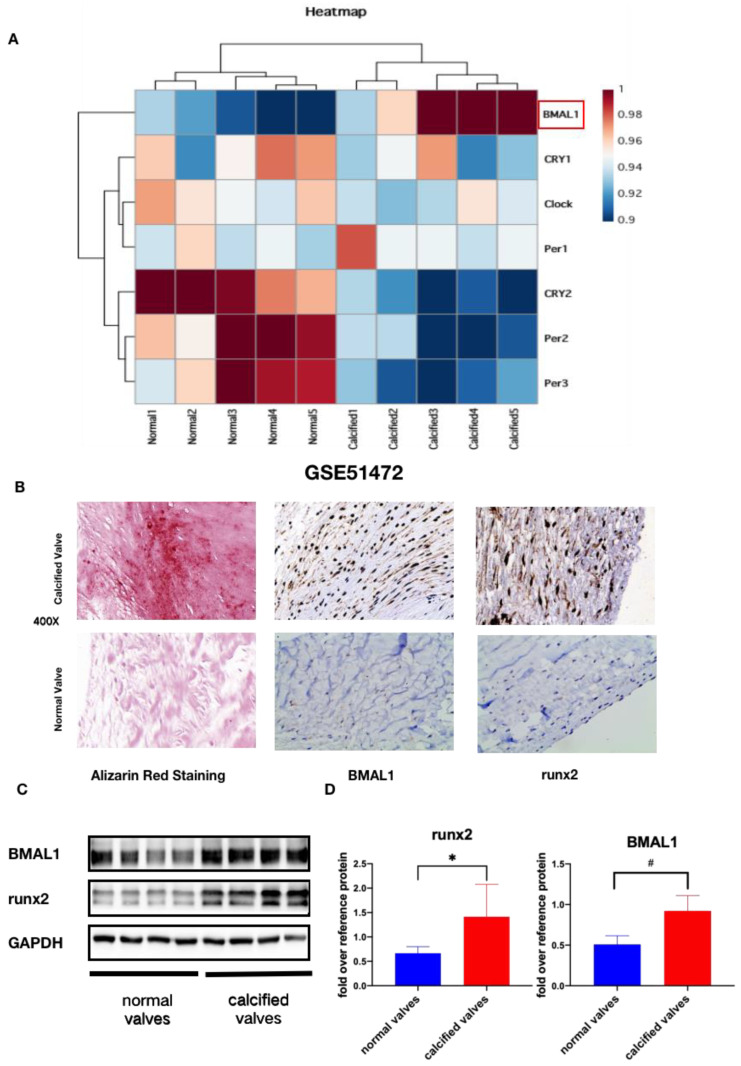
The expression of BMAL1 is increased in human calcified valves. (**A**,**B**) representative images of immunohistochemical staining of BMAL1 and runx2 in normal and calcified valves. (**C**,**D**) the protein levels of BMAL1 and runx2 were increased in calcified valves compared with normal valves (normal valves, *n* = 7; calcified valves, *n* = 8; * *p* < 0.05, # *p* < 0.01).

To confirm our hypothesis, we analyzed the gene expression profiles of normal and calcified valves from GSE51472 and found that calcified valves displayed higher expression of BMAL1 (Figure 1A). Furthermore, we collected normal and calcified valves and immunohistochemical staining and Western blotting were applied to determine BMAL1 expression. The patients’ data are summarized in Table 1; no significant differences were observed between the two groups. Alizarin red staining, immunohistochemical staining and Western blotting showed that—compared with normal valves—with the expression of runx2 or deposition of calcium increased in calcified valves, BMAL1 expression was also increased (Figure 1B–D). These results suggested that BMAL1 might play an important role in CAVD.

### 2.2. BMAL1 Is Associated with Osteogenic Differentiation of HVICs

**Figure 2:**
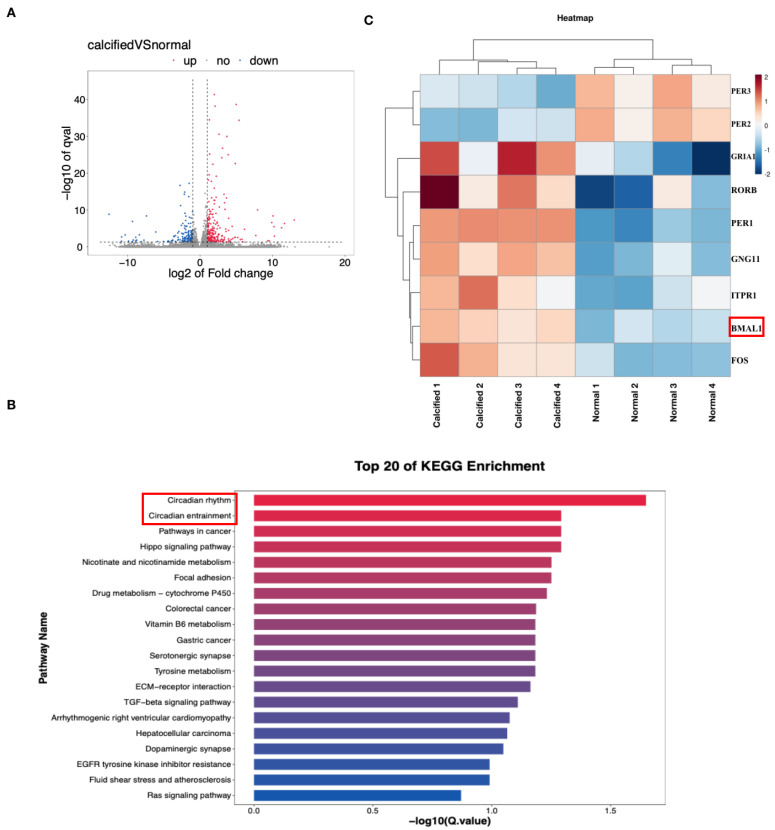
RNA seq of HVICs treated with normal and osteogenic medium. (**A**) gene expression profile with RNA-seq of HVICs treated with normal and osteogenic medium, where 221 upregulated genes and 143 downregulated genes were detected; (**B**,**C**) circadian rhythm and entrainment were top two pathways of KEGG enrichment and BMAL1 was one of the related genes.

**Figure 3:**
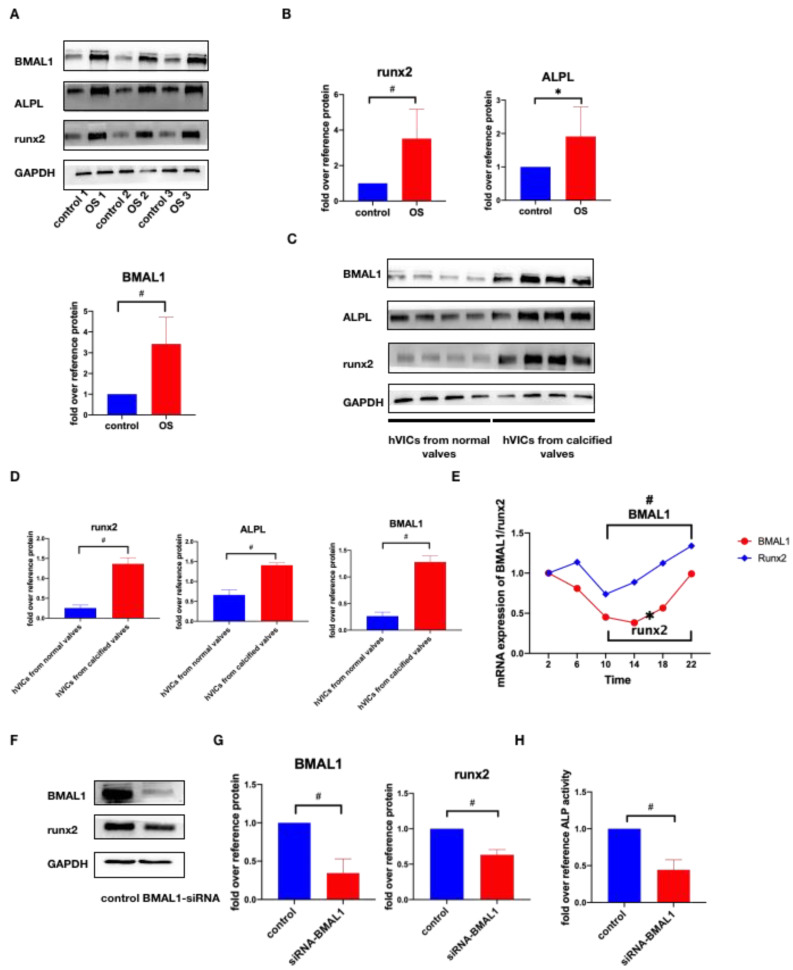
BMAL1 is associated with osteogenic differentiation of HVICs. (**A**,**B**) osteogenic medium could promote BMAL1, runx2 and ALPL expression in HVICs, with *n* = 6 for each group. (**C**,**D**) BMAL1 expression was increased in HVICs isolated from calcified valves compared with HVICs isolated from normal valves, with *n* = 4 for each group. (**E**) runx2 expression showed circadian rhythms synchronizing with BMAL1 expression, with *n* = 4 for each group. (**F**,**G**) after silencing BMAL1, runx2 expression was also reduced, with *n* = 3 for each group. (**H**) after silencing BMAL1, ALP activity was also reduced, with *n* = 6 for each group. * *p* < 0.05, # *p* < 0.01, OS, osteogenic medium.

Since HVICs undergo phenotype differentiation into osteoblast-like cells in CAVD, and the phenotype change of HVICs is the main cause of CAVD, we further investigated whether BMAL1 was associated with HVICs’ osteogenic differentiation. Firstly, we incubated HVICs with osteogenic medium for 7 days to stimulate osteogenic differentiation (OS group) and analyzed the gene expression profiles of HVICs from the control group (normal medium) and OS group by RNA-seq. In total, 221 upregulated genes and 143 downregulated genes were detected (Figure 2A). KEGG analysis showed that the circadian rhythm and entrainment were the top two pathways (Figure 2B). Among the typical circadian-rhythm- and entrainment-related genes, nine genes displayed a change greater than two-fold with *p* < 0.05, and BMAL1 was one of them (Figure 2C). Secondly, we incubated HVICs with osteogenic medium for 3 days (OS group) and checked the protein expression of BMAL1. The protein level of BMAL1 was higher in the OS group (Figure 3A,B). Thirdly, we identified the protein expression of HVICs isolated from normal and calcified valves and found that BMAL1 expression was upregulated in HVICs isolated from calcified valves (Figure 3C,D). Fourthly, to check whether runx2 expression showed circadian rhythms synchronizing with BMAL1 expression, we detected the mRNA expression of runx2 from 2:00 A.M. to 22:00 P.M. every 4 h, and the result demonstrated that runx2 expression showed circadian rhythms synchronized with BMAL1 expression (Figure 3E). To confirm whether BMAL1 was the critical point in the osteogenic differentiation of HVICs, we silenced BMAL1 in HVICs and checked the protein expression of runx2 and alp activity. We found that, after silencing BMAL1, the protein level of runx2 and alp activity decreased (Figure 3F–H). Thus, BMAL1 is associated with the osteogenic differentiation of HVICs and is the key point in this process.

### 2.3. Osteogenic Medium Promotes BMAL1 Expression through TGF-β/RhoA/ROCK Pathway

**Figure 4:**
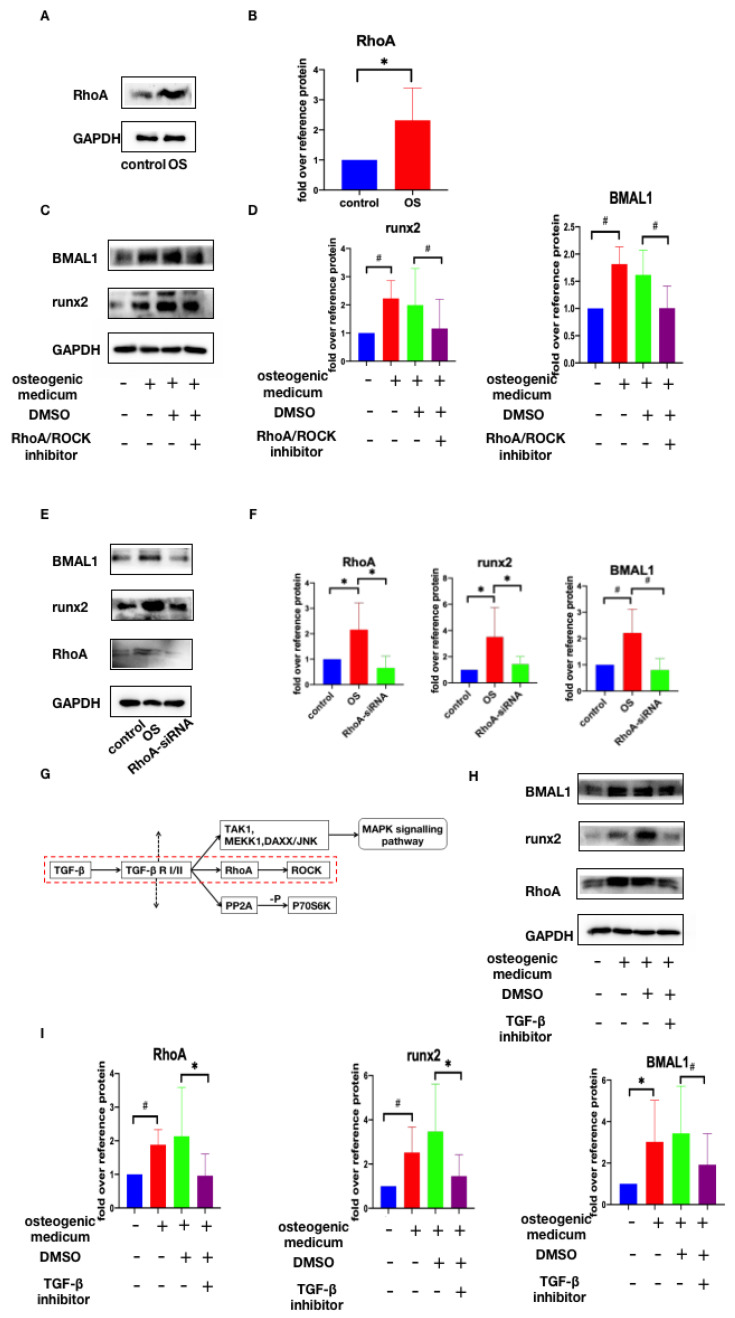
Osteogenic medium promotes BMAL1 expression through TGF-β/RhoA/ROCK pathway. (**A**,**B**) RhoA expression was increased in osteogenic medium, with *n* = 6 for each group. (**C**–**F**) RhoA/Rock inhibitor and RhoA-siRNA could both prevent osteogenic medium from promoting BMAL1 expression, with *n* = 6 for each group. (**G**–**I**) RhoA/ROCK was involved in TGF-β signaling pathway and TGF-β inhibitor could reduce RhoA and BMAL1 expression in osteogenic medium, with *n* = 6 for each group. * *p* < 0.05, # *p* < 0.01, OS, osteogenic medium.

A previous study reported that the RhoA/ROCK pathway could transduce signals provided by extracellular stiffness into cells and regulate the activity of the core circadian clock complex [32]. We hypothesized that RhoA/ROCK was also involved in the osteogenic medium promoting BMAL1 expression. We incubated HVICs in normal and osteogenic medium, and Western blotting showed that RhoA expression was increased in osteogenic medium (Figure 4A,B). Further, RhoA/ROCK inhibitor Y-27632 (10 μM) from Selleck and RhoA-siRNA was added separately. Western blotting showed that after the RhoA/ROCK inhibitor and RhoA-siRNA were added, BMAL1 expression was reduced (Figure 4C–F). As Figure 2A shows, the TGF-β signaling pathway was one of the top 20 pathways of KEGG enrichment. Moreover, the RhoA/ROCK pathway is also included in the TGF-β signaling pathway (Figure 4G). We considered whether this signaling pathway was involved in the osteogenic medium promoting BMAL1 expression. TGF-β inhibitor SB525334 (10 μM) from Selleck was added and Western blotting showed that the protein level of BMAL1 was lower after its addition (Figure 4H,I). Moreover, RhoA expression was also decreased (Figure 4H,I). Therefore, TGF-β/RhoA/ROCK was the key signaling pathway in the osteogenic medium promoting BMAL1 expression.

### 2.4. BMAL1 Cannot Act as a Transcription Factor in Regulating Osteogenic Differentiation of HVICs

**Figure 5:**
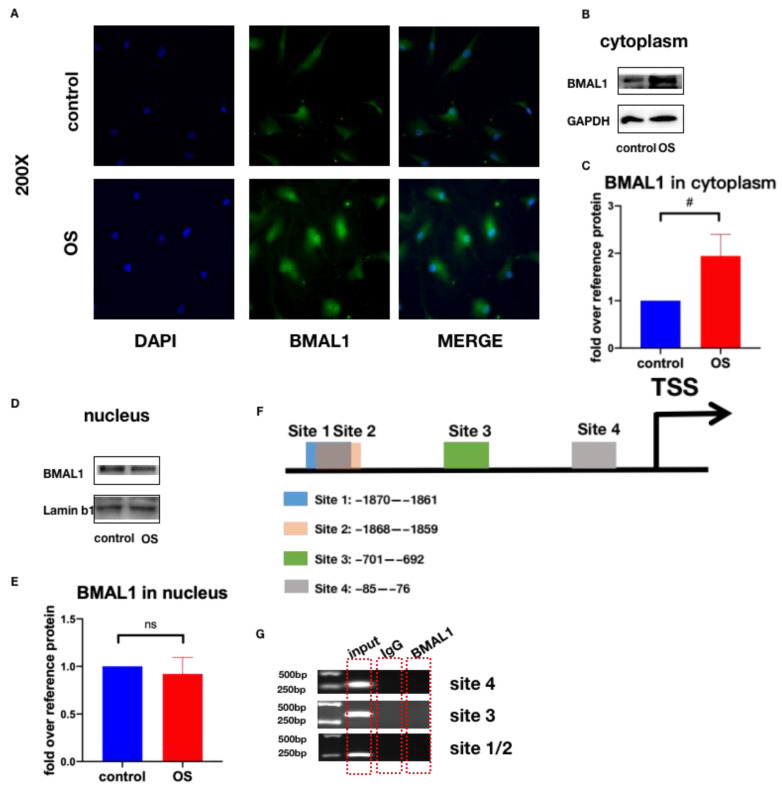
BMAL1 cannot act as a transcription factor in regulating osteogenic differentiation of HVICs. (**A**) representative images of immunofluorescence staining of BMAL1 in HVICs in normal and osteogenic medium. (**B**,**C**) BMAL1 expression was increased in cytoplasm when HVICs were incubated with osteogenic medium, with *n* = 6 for each group. (**D**,**E**) no changes in the protein expression of BMAL1 in nucleus could be found when HVICs were incubated with osteogenic medium, with *n* = 6 for each group. (**F**) 4 predicted binding sites on runx2 promoter CPG region using JASPAR database. (**G**) ChIP detection of four possible binding sites, and BMAL1 could not bind with runx2 promoters. # *p* < 0.01, OS, osteogenic medium.

Considering that BMAL1 can serve as a transcription factor alone or combined with CLOCK to form heterodimers that can activate the transcriptional activity of downstream genes, we needed to determine whether BMAL1 could act as a transcription factor here [24,26,27]. HVICs were incubated in normal and osteogenic medium as the control and OS group separately. Firstly, we checked whether osteogenic medium could facilitate the movement of BMAL1 into the nucleus via immunofluorescence staining. The results showed that BMAL1 existed in both the cytoplasm and nucleus and BMAL1 expression was increased in the cytoplasm in the OS group compared with the control group (Figure 5A). Secondly, we checked the protein level of BMAL1 in the cytoplasm and nucleus. Western blotting showed that more BMAL1 existed in the cytoplasm when HVICs were incubated with osteogenic medium, and no significant difference existed in the protein expression of BMAL1 in the nucleus between the control and OS group (Figure 5B–E). Thirdly, we used the JASPAR database to predict the possible binding sites of BMAL1 on the runx2 promoter CPG region. Four possible binding sites were detected (Figure 5F). Lastly, we determined whether BMAL1 could bind with runx2 promoters through ChIP. Although four possible binding sites were detected, BMAL1 could not bind with runx2 promoters directly (Figure 5G). Taken together, these results suggested that BMAL1 could not act as a transcription factor in regulating the osteogenic differentiation of HVICs.

### 2.5. BMAL1 Promotes HVIC Osteogenic Differentiation through NF-κ B/AKT/MAPK Pathway

**Figure 6:**
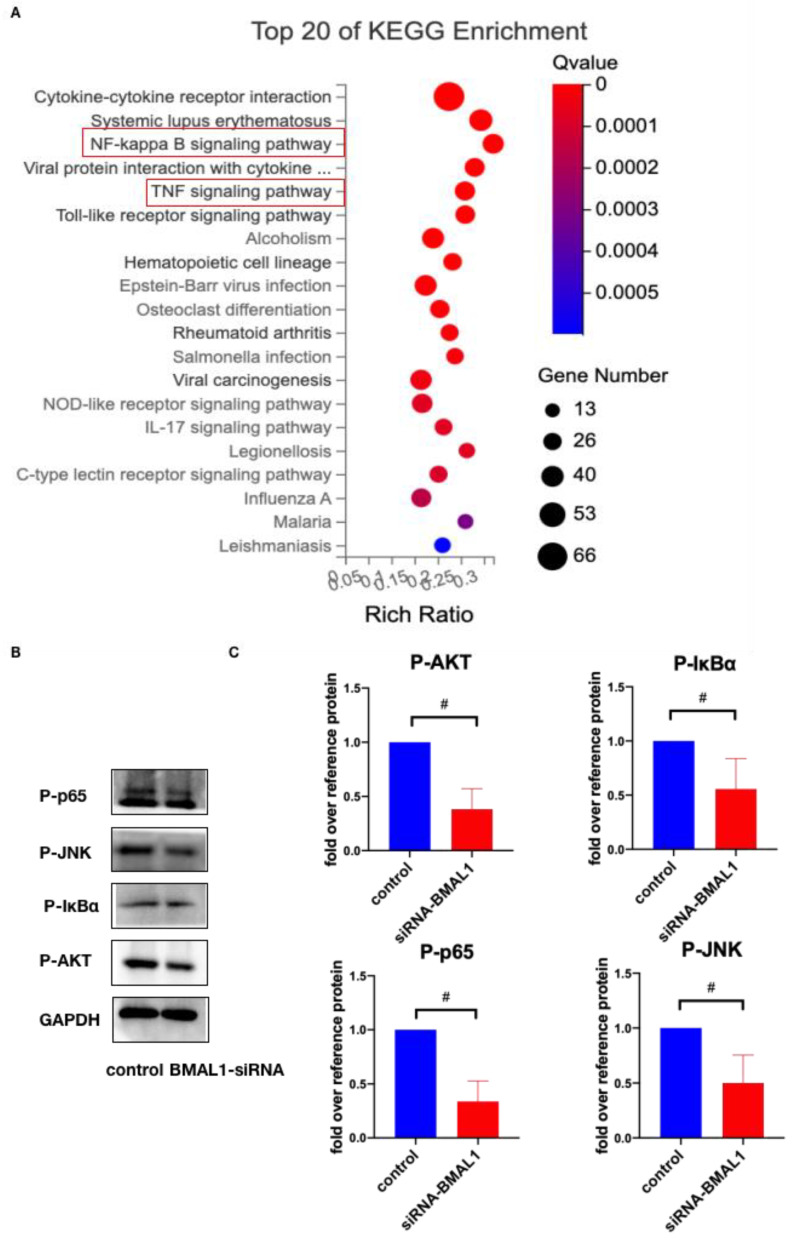
BMAL1 promotes HVICs’ osteogenic differentiation through NF-κ B/AKT/MAPK pathway. (**A**) gene expression profile with RNA seq of HVICs treated with and without BMAL1-siRNA. TNF signaling pathway and NF-κ B pathway were among top 20 pathways of KEGG enrichment. (**B**,**C**) protein levels of phosphorylated AKT, IκBα, p65 and JNK were decreased in HVICs treated with BMAL1-siRNA, with *n* = 6 for each group. # *p* < 0.01.

Following the silencing of BMAL1 in HVICs, substantial differences in gene expression were observed versus the control group. KEGG pathway enrichment analysis was applied and the TNF signaling pathway and NF-κ B pathway were among the top 20 pathways in KEGG enrichment (Figure 6A). Further, the protein levels of phosphorylated AKT, IκBα, p65 and JNK were checked. Western blotting showed that the protein levels of P-AKT, P-IκBα, P-p65 and P-JNK were decreased after BMAL1 was silenced (Figure 6B,C). These results provide an indicator that BMAL1 may promote HVICs’ osteogenic differentiation through the NF-κ B/AKT/MAPK pathway.

## 3. Discussion

CAVD is a disease threatening many people’s health, and the osteogenic differentiation of HVICs is the main cause. In this study, we found that BMAL1 was associated with CAVD. Compared with normal valves, BMAL1 expression was elevated in calcified valves. Additionally, BMAL1 can enhance HVICs’ osteogenic differentiation. Osteogenic medium could promote BMAL1 expression in HVICs through the TGF-β/RhoA/ROCK pathway, and knockdown of BMAL1 resulted in a reduced protein level of runx2. Moreover, the NF-κ B/AKT/MAPK pathway was involved in the signaling mechanism for the osteogenic differentiation of HVICs that was induced by BMAL1. These data offer novel insights into the pathogenesis of CAVD.

The circadian clock genes were first discovered in 1994 [18]. In vertebrates, clock genes can mediate circadian rhythms through a transcription-translation-based autoregulatory feedback loop [33]. Circadian rhythms have been implicated in various physiological processes [6]. Disturbed circadian rhythms could lead to diseases such as cancer [34]. Previous studies have reported that circadian rhythms may be associated with calcification. The incidence of osteoporosis and fracture is higher among shift workers and a short duration of sleep increased the relative risk of coronary artery disease [9,35]. Animal models confirmed that atherosclerosis and vascular calcification were aggravated in mice with disturbed circadian rhythms [21,36]. In our study, we found that the mRNA level of runx2 in HVICs showed 24 h periodicity, which offers an indication that CAVD development may be associated with circadian rhythms and disordered day and night rhythms might result in the progression of CAVD. Of course, further investigation is still needed.

BMAL1 is one of the clock genes and is related to tumors, metabolic diseases, aging, etc. [18,37,38]. BMAL1^−/−^ mice lost circadian rhythmicity and displayed decreased activity, body weight and longevity [31]. Moreover, BMAL1^−/−^ mice also had pathological changes related to calcification, such as tendon calcification and a low bone mass phenotype [19,31]. In our work, we did not study the role of BMAL1 in SCN but in aortic valves and circadian rhythms, which might not be disturbed. Previous studies have confirmed that BMAL1 in peripheral tissues might participate in the calcification process. Mao et al. found that downregulating BMAL1 in bone marrow stromal cells played an inhibitory role in osteogenic differentiation [30]. Liu et al. showed that knockdown of BMAL1 in OCCM-30 cells (an immortalized murine cementoblast cell line) resulted in the downregulation of osteogenic markers and reduced formation of mineralized nodules [39]. Our study presented a similar result. The protein level of BMAL1 was increased in calcified valves. HVICs in osteogenic medium displayed the upregulation of BMAL1 and osteogenic markers. After silencing BMAL1, the osteogenic differentiation of HVICs was inhibited. These results proved that BMAL1 is a key point during HVICs’ osteogenic differentiation.

In SCN, BMAL1 acts as a central clock gene and is regulated by the light/dark cycle, food intake and activity [40]. However, in HVICs, it serves as a peripheral clock gene. Although peripheral clock genes can be regulated by central clock genes, they are also regulated by the cellular microenvironment [41]. For example, Williams et al. reported that clock genes in epithelial and stromal cells can be regulated by their mechano-matrix environment [42]. In our study, RNA-seq of HVICs in control and osteogenic medium showed that the circadian rhythm and entrainment were the first 2 pathways among the top 20 pathways of KEGG enrichment, which supports our hypothesis that HVICs can sense the osteogenic medium and adapt to it. The fact that the protein level of BMAL1 was also increased in HVICs in osteogenic medium supports our hypothesis further. Yang et al. reported that the RhoA/ROCK pathway transduced signals provided by extracellular stiffness into cells and regulated the activity of the circadian clock complex [32]. We hypothesized that the RhoA/ROCK pathway was also involved in the osteogenic medium promoting BMAL1 expression in HVICs. To verify our hypothesis, a RhoA/ROCK inhibitor and RhoA-siRNA were applied and the osteogenic medium promoting BMAL1 expression was inhibited. In total, 3 signaling pathways were among the top 20 pathways of KEGG enrichment; only the TGF-β signaling pathway contained the RhoA/ROCK pathway. To check the function of the TGF-β pathway here, we added the TGF-β inhibitor and the osteogenic medium promoting BMAL1 expression was also inhibited. These data confirmed that the osteogenic medium could promote HVICs’ osteogenic differentiation through the TGF-β/RhoA/ROCK signaling pathway.

BMAL1 can serve as a transcription factor alone or combined with CLOCK to form heterodimers, which can activate the transcriptional activity of downstream genes [24,26,27]. It is a basic helix-loop-helix PAS domain transcription factor that exerts its function by binding to the E-box elements of CACGTG-type (or CACGTT-type-like) in the promoters of its downstream target genes. Zhou et al. showed that BMAL1 could bind directly to the Opg promoter and upregulate its expression [43]. Min et al. reported that BMAL1 was a direct regulator of insulin-mediated osteoblast differentiation by increasing the promoter activity of BMP2 in MC3T3-E1 cells [44]. In our study, JASPAR database analysis showed four potential BMAL1 binding sites on the runx2 promoter CPG region. However, ChIP showed that BMAL1 could not bind with runx2 promoters directly. Moreover, immunofluorescence and Western blotting showed that more BMAL1 existed in the cytoplasm only when HVICs were treated with osteogenic medium. These data confirmed that BMAL1 could not act as a transcription factor in regulating runx2 expression. RNA-seq of HVICs treated with and without BMAL1-siRNA showed that the TNF signaling pathway and NF-κ B pathway were among the top 20 pathways of KEGG enrichment, and Western blotting confirmed that the protein levels of P-AKT, P-IκBα, P-p65 and P-JNK were decreased after BMAL1 was knocked down. We concluded that BMAL1 promoted HVICs’ osteogenic differentiation through the NF-κ B/AKT/MAPK pathway.

## 4. Materials and Methods

### 4.1. Patient Selection and Specimen Acquisition

The protocol of this study was approved by the Ethical Committee of Jiangsu Province Hospital, affiliated to Nanjing Medical College, China, and conducted in accordance with the ethical standards stated in the Declaration of Helsinki. Informed consent was obtained from all patients before surgery. Calcified aortic valves were obtained from 50 patients with CAVD who underwent aortic valve replacement. Control normal aortic valves were collected from 50 age-matched patients who underwent heart transplant or Bentall procedures (acute aortic dissection).

### 4.2. Cell Culture and Treatment

HVICs were obtained from normal and calcified valves using collagenase I digestion, as described previously [4]. Interstitial cells were cultured in Dulbecco’s modified Eagle medium containing 1% penicillin G and streptomycin and 10% fetal bovine serum at 37 °C and 5% CO_2_. The cells from passages 3 to 5 were used for the following experiments and incubated in osteogenic medium to stimulate osteogenic differentiation, as previously described [45]. Here, osteogenic medium contained 1% fetal bovine serum, 50 mg/mL ascorbic acid, 100 nmol/L dexamethasone and 10 mmol/L β-glycerophosphoric acid.

### 4.3. Immunohistochemistry

Aortic valves were harvested, rinsed with cold phosphate-buffered saline, fixed in 4% paraformaldehyde and embedded in paraffin. Immunohistochemical staining was performed, as previously described, with the following antibodies: BMAL1 (Proteintech, 1:200), runx2 (Proteintech, 1:200) [4].

### 4.4. Immunofluorescence Staining

HVICs were fixed with 4% paraformaldehyde for 10–20 min, and subsequently permeabilized with 0.5% Triton-X-100 for 10 min. Then, the cells were treated with BMAL1 (Proteintech, 1:200) overnight at 4 °C. Primary antibodies were removed and fluorescent-conjugated secondary antibody (Proteintech, 1:5000) was added. Images were taken with a fluorescence microscope (Leica).

### 4.5. Western Blotting

Protein expression was demonstrated by Western blotting, following instructions described previously [4]. Moreover, cell cytoplasm and nucleus proteins were separated using kits from Beyotime. The following antibody dilutions were used: BMAL1 (Proteintech, 1:500), runx2 (CST, 1:1000), alkaline phosphatase (ALP) (R&D, 1:500), gapdh (Proteintech, 1:1000), Lamin B1 (Proteintech, 1:1000), phospho-JNK MAPK (cst, 1:1000), phospho-NF-kB (cst, 1:500), phospho-AKT (cst, 1:1000), phospho-IκBα (cst, 1:500) and phospho-p65 (cst, 1:1000).

### 4.6. Real-Time Polymerase Chain Reaction RNA Analysis

RNA of cells was isolated as previously described [4]. Real-time reverse transcription polymerase chain reaction (RT-PCR) assays were carried out using the One Step qRT-PCR Probe Kit from Vazyme. Primers were as follows: BMAL1 (F: 5′-TGCCCTCTGGAGAAGGTGG-3′; R: 5′-GGAGGCGTACTCGTGATGTT-3′); runx2 (F: 5′-TCGCCTCACAAACAACCACA-3′; R: 5′-GCTTGCAGCCTTAAATGACTCT-3′); and gapdh (F: 5′-CATGTTCGTCATGGGTGTGAACCA; R: 5′-AGTGATGGCATGGACTGTGGTCAT-3′). Results were normalized to Gapdh and the delta/delta CT calculation method was used to analyze the data.

### 4.7. Chromatin Immunoprecipitation (ChIP)

ChIP assays were conducted using the Chromatin Immunoprecipitation Kit (Millipore), following the manufacturer’s protocol. Briefly, cells were crosslinked and lysed. DNA fragments less than 500 bp were prepared by sonication in ChIP dilution buffer. Proteins were immunoprecipitated in ChIP dilution buffer using BMAL1 antibodies (1:250) and then incubated overnight at 4 °C. Crosslinking was reversed at 65 °C in elution buffer (50 mmol/L Tris–HCl, 10 mmol/L EDTA and 1% SDS, pH 8.0) for 5 h, after which DNA was isolated. PCR was performed using primers specific to the BMAL1 hypersensitive site on the runx2 promoters. The primer sequences used are shown in Table S1. Furthermore, agarose gel electrophoresis was applied.

### 4.8. Silencing BMAL1 or RhoA

To knock down BMAL1 or RhoA, cells (80% confluence) in 6-well plates were incubated with a small interfering RNA (siRNA) (50 nmol/L), using Lipofectamine 3000 (Invitrogen) and Opti-Men (Life Technologies), according to the manufacturer’s instructions. Meanwhile, control cells were treated with scrambled siRNA. The medium was changed 12 h after transfection; 48 h later, the cells were harvested for protein expression analysis. The sequence of BMAL1 siRNA is TCACCAAGATGACATAGGA, and the sequence of RhoA siRNA is AGAACTATGTGGCAGATAT.

### 4.9. Detection of mRNA Profiles

RNA-sequencing (RNA-seq) quantification was utilized to investigate changes in cell mRNA profiles among different treatments performed. Isolated RNA was sequenced by BGI Co., Ltd. and LC-Bio Technology Co., Ltd. Sequencing results were further analyzed in order to identify differentially expressed genes (DEGs) and perform Kyoto Encyclopedia of Genes and Genomes (KEGG) pathway enrichment analysis using the R language.

### 4.10. Alizarin Red Staining

Alizarin red staining for cell calcium deposits was performed as described previously [4]. For tissues, deparaffinized sections were incubated with alizarin red solution for 5–10 min, and excess dye was removed by washing with distilled water.

### 4.11. Alkaline Phosphatase (ALP) Activity

ALP activity in the cell lysates was assayed using a colorimetric assay kit (Beyotime) by measuring the p-nitrophenol release in absorbance at 405 nm. Results are presented as relative ALP activity normalized to that of the control cells.

### 4.12. Statistical Analysis

Continuous data were expressed as mean ± standard deviation. The differences between 2 groups were assessed by Student’s *t*-test. Categorical data were expressed as a percentage and compared with the chi-square test. Statistical analysis was carried out using SPSS 26.

## 5. Study Limitation

Several limitations exist in our study. First, only in vitro experiments were conducted in this study to elaborate the role of BMAL1 in HVICs’ osteogenic phenotype. Whether BMAL1 promotes CAVD progression in preclinical animal models still requires further investigations. Second, calcification markers were limited in this study. Third, BMAL1 is a circadian clock gene; whether disturbed circadian rhythms will result in CAVD development still needs additional investigation.

## 6. Conclusions

In summary, we examined the role of BMAL1 in promoting HVICs’ osteogenic differentiation. This study provides us with an important potential therapeutic target and a strong rationale for CAVD.

## Figures and Tables

**Table 1:** Clinical characteristics of selected patients.

	Normal (*n* = 7)	Calcified (*n* = 8)	*p* Value
Age	47.29 ± 8.66	58.25 ± 15.70	0.13
Male	7	6	0.47
Hypertension	2	2	1
Diabetes	3	0	0.08
BMI	25.80 ± 2.64	23.74 ± 2.61	0.16
LDL	2.13 ± 1.01	2.75 ± 0.89	0.23
HDL	1.26 ± 0.42	1.15 ± 0.35	0.56
Triglycerides	1.43 ± 0.71	1.03 ± 0.34	0.18
Creatinine	93.91 ± 20.08	75.45 ± 15.06	0.06

BMI: body mass index; LDL: low-density lipoprotein; HDL: high-density lipoprotein.

## Data Availability

The data presented in this study are available on request from the corresponding author.

## Supplementary Materials

The following supporting information can be downloaded at: https://www.mdpi.com/article/10.3390/jcdd10030110/s1, Table S1: Primer sequences used in Chip.
