# Supplementary material for: BMAL1 Promotes Valvular Interstitial Cells’ Osteogenic Differentiation through NF-κ B/AKT/MAPK Pathway
Source: J Cardiovasc Dev Dis. 2023 Mar 6;10(3):110. doi: 10.3390/jcdd10030110 (PMC10054744; doi:10.3390/jcdd10030110)
Supplement: Supplementary file 1 [file jcdd-10-00110-s001.zip › jcdd-2228951-supplementary.pdf]

Supplementary Table S1

		Primer sequences(5'to3')
Binding site 1/2	F	GGTAGGCAGTCCCACTTTACTTAA
	R	ACTGACTCTGTTGGTCTCGGTG
Binding site 3	F	CCCTCCTTTTGTTTACTTTGGA
	R	CACTGGTAGGGTATGGGAATGA
Binding site 4	F	CTTCAAACTAGGCATGAGATAATGG
	R	GGTGGCTGGATTCCTTCTGTT

Supplementary Table S1: Primer sequences used in Chip.
